# Discovery and genetic characterization of diverse smacoviruses in Zambian non-human primates

**DOI:** 10.1038/s41598-019-41358-z

**Published:** 2019-04-08

**Authors:** Paulina D. Anindita, Michihito Sasaki, Gabriel Gonzalez, Wallaya Phongphaew, Michael Carr, Bernard M. Hang’ombe, Aaron S. Mweene, Kimihito Ito, Yasuko Orba, Hirofumi Sawa

**Affiliations:** 1 Division of Molecular Pathobiology, Research Center for Zoonosis Control, Hokkaido University, Sapporo, 001-0020 Japan; 2 Division of Bioinformatics, Research Center for Zoonosis Control, Hokkaido University, Sapporo, 001-0020 Japan; 3 Global Institution for Collaborative Research and Education (GI-CoRE), Hokkaido University, Sapporo, 001-0020 Japan; 4 National Virus Reference Laboratory, School of Medicine, University College Dublin, Belfield, Dublin, 4 Ireland; 5 Department of Paraclinical Studies, School of Veterinary Medicine, University of Zambia, PO Box 32379, Lusaka, 10101 Zambia; 6 Department of Disease Control, School of Veterinary Medicine, University of Zambia, PO Box 32379, Lusaka, 10101 Zambia; 7 Africa Centre of Excellence for Infectious Diseases of Humans and Animals, University of Zambia, P.O. Box 32379, Lusaka, 10101 Zambia; 8 Global Virus Network Affiliate, University of Zambia, P.O. Box 32379, Lusaka, 10101 Zambia; 9 Global Virus Network, 801 W. Baltimore St., Baltimore, MD 21201 USA

**Keywords:** Viral evolution, Phylogenomics, Viral evolution, Phylogenomics

## Abstract

The *Smacoviridae* has recently been classified as a family of small circular single-stranded DNA viruses. An increasing number of smacovirus genomes have been identified exclusively in faecal matter of various vertebrate species and from insect body parts. However, the genetic diversity and host range of smacoviruses remains to be fully elucidated. Herein, we report the genetic characterization of eleven circular replication-associated protein (*Rep*) encoding single-stranded (CRESS) DNA viruses detected in the faeces of Zambian non-human primates. Based on pairwise genome-wide and amino acid identities with reference smacovirus species, ten of the identified CRESS DNA viruses are assigned to the genera *Porprismacovirus* and *Huchismacovirus* of the family *Smacoviridae*, which bidirectionally encode two major open reading frames (ORFs): *Rep* and capsid protein (*CP*) characteristic of a type IV genome organization. The remaining unclassified CRESS DNA virus was related to smacoviruses but possessed a genome harbouring a unidirectionally oriented *CP* and *Rep*, assigned as a type V genome organization. Moreover, phylogenetic and recombination analyses provided evidence for recombination events encompassing the 3′-end of the *Rep* ORF in the unclassified CRESS DNA virus. Our findings increase the knowledge of the known genetic diversity of smacoviruses and highlight African non-human primates as carrier animals.

## Introduction

Recent advances in high-throughput sequencing technologies have allowed metagenomic analyses to discover an ever-increasing genetic diversity of viral genomes from vertebrates, invertebrates, prokaryotic and environmental samples^1^. Small circular replication-associated protein (*Rep*) encoding single-stranded (CRESS) DNA viruses have been discovered in a diversity of prokaryotes and eukaryotes^2^. With the increasing diversity of CRESS DNA viruses, they have been classified into six viral families: *Genomoviridae*, *Geminiviridae*, *Nanoviridae*, *Circoviridae*, *Bacilladnaviridae* and *Smacoviridae*. The family *Smacoviridae* was recently assigned as a new viral family by the International Committee on Taxonomy of Viruses (ICTV)^3^, which is further classified into six genera. Smacoviruses have 2.3–2.9 kb genomes, containing two major open reading frames (ORFs), encoding *Rep* and the capsid protein (*CP*). Rep possesses DNA helicase activity and initiates viral replication by a rolling circle replication (RCR)^4^. In comparison with *Rep*, the *CP* ORF is more divergent among smacoviruses. The genetic diversity observed within smacoviruses might be due to high mutation rates^5^ and intra-familial recombination events in their genomes^6^.

Smacoviruses, previously known as “stool-associated circular viruses”, have been detected in faecal samples obtained from healthy and diarrheic animal species, including cattle^7^, sheep^7^, pigs^8,9^, rats^10^, chickens^11^, camels^12^, non-human primates^13^ and humans^13–15^, as well as insect species such as dragonflies^16^ and blow flies^17^ but not from environmental samples. Despite the lack of evidence for a direct causal relationship, smacoviruses were identified in the faecal virome derived from human patients with diarrhea in France as well as in central and south American children with unexplained gastrointestinal disease negative for known pathogens^13,15^. It remains, however, to be established whether smacovirus infect human cells, causes overt disease or not in humans and animals.

In this study, sequence reads related to CRESS DNA virus genomes were initially discovered in faecal samples of Zambian NHPs through viral metagenomic analysis. We subsequently determined whole genome sequences of eleven CRESS DNA viruses and characterized ten of them as new smacovirus species. This study extends the known genetic diversity of smacoviruses and the species range of NHPs which harbour these ssDNA viruses.

## Results

### Identification of CRESS DNA viruses in Zambian NHP species

Fifty faecal samples from NHPs consisting of 25 malbroucks (*Chlorocebus cynosuros*) and 25 baboons (*Papio spp*.) in Zambia were suspended, pooled and subjected to metagenomic analysis. Among a total of 63,587,648 sequence reads generated, 1,381,545 reads were assigned to ssDNA viruses by BLASTx by comparison of the translated nucleotide sequences from the samples with the viral protein database^18^. Six contigs, ranging in length from 0.8–2.0 kb, related to members of *Smacoviridae* were generated by *de novo* assembly.

To examine the prevalence of the smacovirus-like genomes, six different pairs of primer sets were designed and used to screen fifty faecal samples from NHPs (Supplementary Table S1). Ten (20%) of the NHP faecal samples were positive for smacovirus-like genomes. Nine faecal samples were positive for a single smacovirus-like genome, whereas one malbrouck faecal sample (ZM09#96) harboured two different smacovirus-like genomes (Table 1). Speciation of NHPs was confirmed by sequencing of mitochondrial *cytochrome b* (*cytb*) (Table 1).

**Table 1:** Sample information and results of PCR screening.

Sample	Non-human primate species (common name)	Corresponding primer	Virus	Abbreviation	Accession number
ZM09#51	*Papio cynocephalus* (yellow baboon)	ssDNAV-3	*Papio cynocephalus* associated smacovirus 3/ZM09-51	PcSmV3-ZM09-51	LC386205
ZM09#64	*P*. *kindae* (Kinda yellow baboon)	ssDNAV-1	*Papio kindae* associated smacovirus-like virus 1/ZM09-64	PkSmV1-ZM09-64	LC386203
ZM09#71	*P*. *cynocephalus* (yellow baboon)	ssDNAV-2	*Papio cynocephalus* associated smacovirus 2/ZM09-71	PcSmV2-ZM09-71	LC386204
ZM09#72	*P*. *cynocephalus* (yellow baboon)	ssDNAV-6	*Papio cynocephalus* associated smacovirus 6/ZM09-72	PcSmV6-ZM09-72	LC386201
ZM09#74	*P*. *cynocephalus* (yellow baboon)	ssDNAV-3	*Papio cynocephalus* associated smacovirus 3/ZM09-74	PcSmV3-ZM09-74	LC386197
ZM09#76	*P*. *kindae* (Kinda yellow baboon)	ssDNAV-3	*Papio kindae* associated smacovirus 3/ZM09-76	PkSmV3-ZM09-76	LC386198
ZM09#83	*Chlorocebus cynosuros* (malbrouck)	ssDNAV-5	*Chlorocebus cynosuros* associated smacovirus 5/ZM09-83	CcSmV5-ZM09-83	LC386200
ZM09#86	*C*. *cynosuros* (malbrouck)	ssDNAV-1	*Chlorocebus cynosuros* associated smacovirus 1/ZM09-86	CcSmV1-ZM09-86	LC386195
ZM09#95	*C*. *cynosuros* (malbrouck)	ssDNAV-4	*Chlorocebus cynosuros* associated smacovirus 4/ZM09-95	CcSmV4-ZM09-95	LC386199
ZM09#96	*C*. *cynosuros* (malbrouck)	ssDNAV-1	*Chlorocebus cynosuros* associated smacovirus 1/ZM09-96	CcSmV1-ZM09-96	LC386196
ZM09#96	*C*. *cynosuros* (malbrouck)	ssDNAV-6	*Chlorocebus cynosuros* associated smacovirus 6/ZM09-96	CcSmV6-ZM09-96	LC386202

The complete, circular genomes from eleven smacovirus-like genomes were then amplified by inverse PCR, cloned into plasmid vectors and then sequenced bidirectionally by a primer walking strategy employing Sanger sequencing. As a result, we found that the complete circular genome sizes ranged from 2488 to 2766 nucleotides, which is within the known range of previously reported CRESS DNA virus genomes. BLASTx analysis showed that these CRESS DNA virus genomes were related to known viruses from the family *Smacoviridae*.

### Classification and genome organization of Zambian NHP CRESS DNA viruses

**Figure 1:**
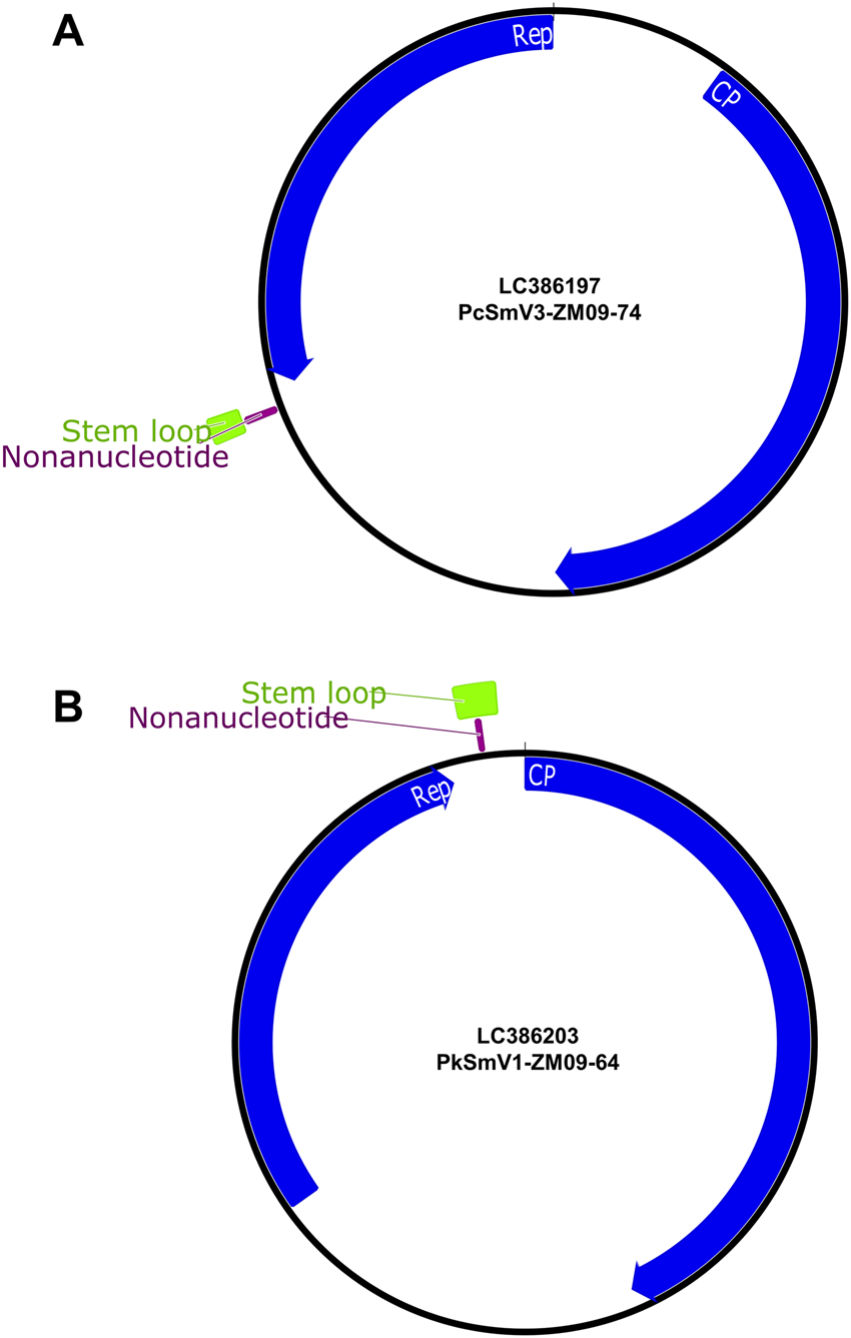
Genome organization of the representative smacovirus carrying a ambisense type IV genome (**A**) and CRESS DNA virus carrying a unisense type V genome (**B**) from Zambian NHPs showing ORFs (*Rep*, rolling replication-associated protein; *CP*, capsid protein), predicted stem loop and nonanucleotide.

The smacovirus-like CRESS DNA viruses from Zambian NHPs each contained two large ORFs which showed sequence similarity to *CP* and *Rep* of previously described smacoviruses. Following the CRESS DNA virus classification scheme proposed by Rosario *et al*.^4^, the genomes of ten CRESS DNA viruses belonged to the ambisense type IV organization whereas the genome of one CRESS DNA virus (isolate PkSmV1-ZM09–64) contained a unisense type V organization in which the *CP* and *Rep* ORFs were in the same orientation similar to the previously described porcine smacovirus-related CRESS DNA virus, PigSCV (JQ023166)^13,19^ (Fig. 1). The predicted stem loop structures were located near the 3′-end of the *Rep* ORF with homology to the degenerate NAGTNTTAC nonanucleotide sequence motif which are also shared by all other reported smacoviruses^6^ (Table 2). This motif has been identified as the putative origin of RCR of smacoviruses during the replication cycle^7,13^.

**Table 2:** Genome features of Zambian non-human primate CRESS DNA viruses.

Virus	Genome length (nt)	Rep (aa)	CP (aa)	Nonanucleotide motif	RCR motif I	RCR motif II	RCR motif III	Walker A	Walker B	Walker C
PkSmV1-ZM09-64	2544	259	368	AGGTCTTAC	ATVWVD	HFQFRG	YVYK	SRGNWGKT	LVDTP	VLCN
CcSmV1-ZM09-86	2691	284	369	AATGATTAC	ATIDR	HWQCRF	YETK	PTGNIGKS	IIDIP	VMTN
CcSmV1-ZM09-96	2659	268	182	AAAAATTAC	VTMGR	HWQVRF	YEAK	ETGNRGKS	VIDIP	VLTN
PcSmV2-ZM09-71	2766	271	359	AATGATTAC	CTVPN	HFQLRC	YVEK	RVGGRGKT	VIDMP	VITN
PcSmV3-ZM09-51	2540	269	312	TAGTGTTAC	MTAPR	HWQCRI	YEAK	ETGNVGKS	IIDVP	VMTN
PcSmV3-ZM09-74	2488	245	330	TAGTATTAC	GTAPR	HWQIRI	YEAK	ETGNVGKS	IIDVP	VMTN
PkSmV3-ZM09-76	2542	269	330	TAGTGTTAC	MTAPR	HWQCRI	YEAK	ETGNRGKS	IIDVP	VMTN
CcSmV4-ZM09-95	2656	260	271	TAGTATTAC	LTIPR	HWQVVV	YCRK	VRGGHGKT	WIDLP	VTTN
CcSmV5-ZM09-83	2617	261	354	TAGTATTAC	GTISA	HYQYTV	YCKK	GGGHGKT	WIDLP	VTTN
PcSmV6-ZM09-72	2613	269	353	TAGTATTAC	GTISA	HFQYYI	YCKK	VGGAHGKT	WIDIP	VTTN
CcSmV6-ZM09-96	2537	269	354	TAGTATTAC	GTISA	HFQYCI	YCKK	VGGAHGKT	WIDLP	VTTN

To further characterize the identified viruses as CRESS DNA viruses, we also searched for amino acid motifs within the encoded Rep that play important functional roles in viral genome replication. All Rep proteins of the identified CRESS DNA viruses harboured an RCR domain (motifs I, II and III) and a helicase super family 3 domain (Walker A, B and C) as illustrated in Table 2. Interestingly, a variation of amino acid residues within the RCR motifs I and II was found throughout the Rep proteins of the identified CRESS DNA viruses compared to the previously described consensus motifs^7^. Notably, ten of eleven CRESS DNA viruses encoded Rep proteins possessing 5 amino acid residues within RCR motif I whereas the Rep of isolate PkSmV1-ZM09-64 had 6 amino acid residues (Table 2). In addition, the *Rep* ORF of PkSmV1-ZM09-64 encoded a leucine residue at the beginning of the Walker B motif which is unseen in smacoviruses, which possess isoleucine, valine or tryptophan at the first residue (Table 2).

The pairwise nucleotide sequence identities were calculated to determine the genetic distances between the identified CRESS DNA virus genomes and previously described smacoviruses (Table 3). The isolate PkSmV1-ZM09-64, carrying a unisense type V genome, was not assigned to a virus family and excluded from this analysis due to the inversion of the replication-associated protein ORF (Fig. 1). All of the identified genomes except PkSmV1-ZM09-64 had <77% genome-wide pairwise nucleotide sequence identity. Based on the smacovirus species demarcation threshold of 77% genome-wide pairwise nucleotide identity^6^, we grouped these CRESS DNA viruses into five smacovirus species (species 2–6 in Table 3). Species 3 and 6 were only identified from *C*. *cynosuros*, whereas species 4 and 5 were identified from 2 different NHP species.

**Table 3:** Pairwise sequence identity among Zambian non-human primate CRESS DNA viruses and known smacoviruses.

Species	Isolate	Closest genome sequence	Identity (%)	Closest genome sequence of known smacovirus species	Identity (%)	Closest Rep sequence of known smacovirus species	Identity (%)	Genus
1	(LC386203) PkSmV1-ZM09-64	*		*		(KT862223) Bovine faeces associated smacovirus 1	42.73	unassigned
2	(LC386204) PcSmV2-ZM09-71	(LC386198) PkSmV3-ZM09-76	56.58	(KP233194) Lemur smacovirus	55.46	(KT862223) Bovine faeces associated smacovirus 1	46.86	*Huchismacovirus*
3	(LC386195) CcSmV1-ZM09-86	(LC386196) CcSmV1-ZM09-96	82.29	(KT600068) Human feces smacovirus 2	64.69	(KT600068) Human feces smacovirus 2	75.18	*Porprismacovirus*
3	(LC386196) CcSmV1-ZM09-96	(LC386195) CcSmV1-ZM09-86	82.29	(KT600068) Human feces smacovirus 2	62.44	(GQ351274) Chimpanzee stool associated circular ssDNA virus	64.48	*Porprismacovirus*
4	(LC386205) PcSmV3-ZM09-51	(LC386198) PkSmV3-ZM09-76	99.80	(KJ577810) Porcine stool associated circular virus 1	67.15	(JX274036) Porcine stool associated circular virus	81.07	*Porprismacovirus*
4	(LC386197) PcSmV3-ZM09-74	(LC386198) PkSmV3-ZM09-76	92.86	(KJ577810) Porcine stool associated circular virus 1	67.84	(JX274036) Porcine stool associated circular virus	83.95	*Porprismacovirus*
4	(LC386198) PkSmV3-ZM09-76	(LC386205) PcSmV3-ZM09-51	99.80	(KJ577810) Porcine stool associated circular virus 1	67.32	(JX274036) Porcine stool associated circular virus	81.48	*Porprismacovirus*
5	(LC386199) CcSmV4-ZM09-95	(LC386201) PcSmV6-ZM09-72	79.62	(KT600068) Human feces smacovirus 2	55.70	(KT862221) Sheep faeces associated smacovirus 2	42.08	*Porprismacovirus*
5	(LC386201) PcSmV6-ZM09-72	(LC386199) CcSmV4-ZM09-95	79.62	(KT600068) Human feces smacovirus 2	55.12	(KT862218) Bovine faeces associated smacovirus 2	39.24	*Porprismacovirus*
6	(LC386200) CcSmV5-ZM09-83	(LC386202) CcSmV6-ZM09-96	80.14	(KT600068) Human feces smacovirus 2	55.00	(KT862218) Bovine faeces associated smacovirus 2	40.92	*Porprismacovirus*
6	(LC386202) CcSmV6-ZM09-96	(LC386200) CcSmV5-ZM09-83	80.14	(KP233194) Lemur smacovirus	56.65	(KT862218) Bovine faeces associated smacovirus 2	39.66	*Porprismacovirus*

*Not comparable due to the inversion of the replication-associated protein ORF.

Pairwise amino acid sequence identity was calculated for the Rep proteins of all smacoviruses from Zambian NHPs (Zm-SmVs) and that of known smacoviruses (Table 3). Among the Zm-SmVs species, four species belonged to the genus *Porprismacovirus* while a single species was most closely related to the genus *Huchismacovirus* following the smacovirus genus demarcation threshold of 40% pairwise amino acid sequence identity of Rep^6^. PcSmV6-ZM09-72 and CcSmV6-ZM09-96 were assigned to the genus *Porprismacovirus* as they were classified as closely-related species to other Porprismacoviruses (CcSmV4-ZM09-95 and CcSmV5-ZM09-83, respectively).

### Phylogenetic relationships among Zambian CRESS DNA viruses and known smacoviruses

**Figure 2:**
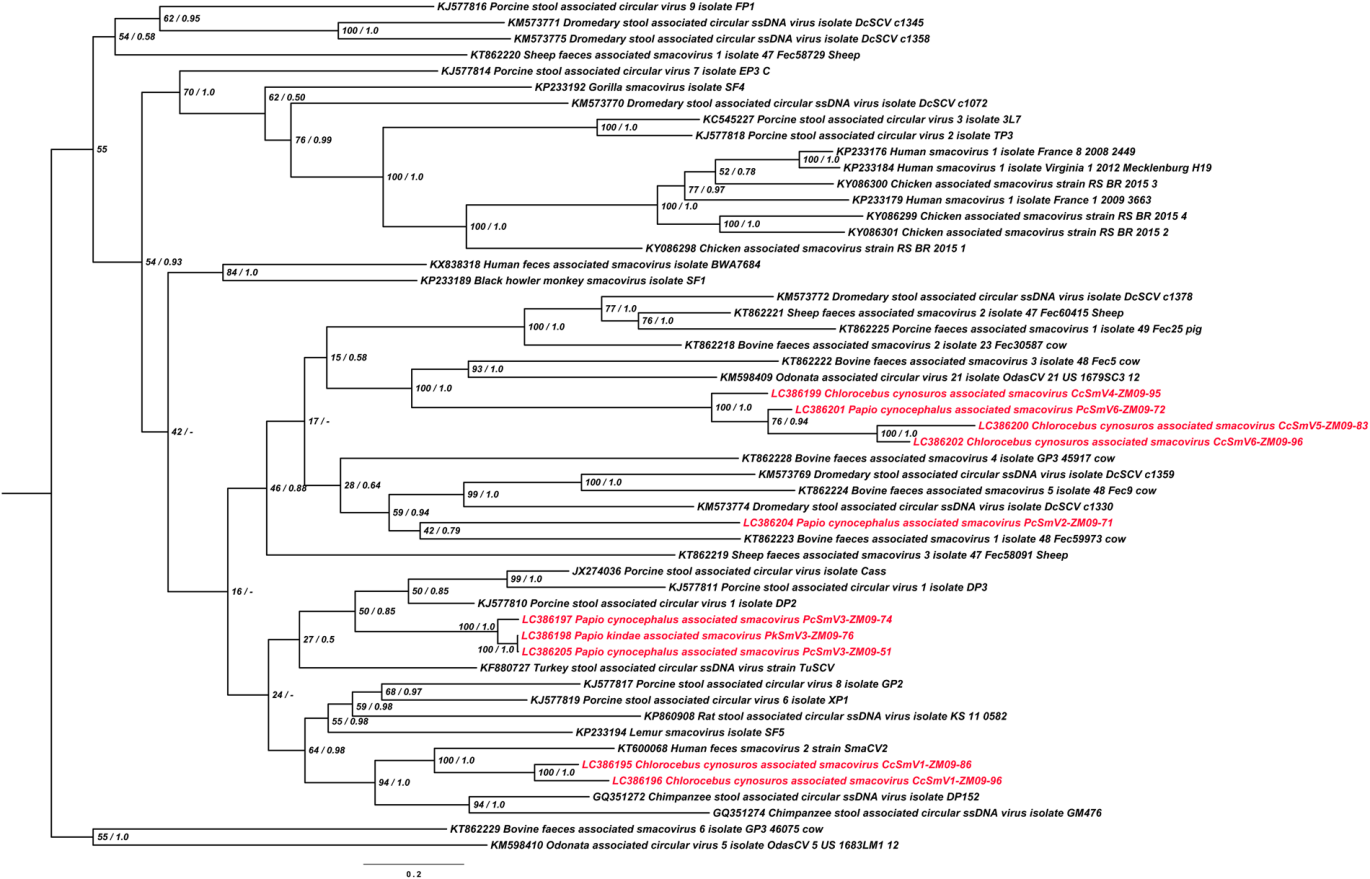
Phylogenetic tree constructed based on the whole genome nucleotide sequences of Zambian non-human primate CRESS DNA viruses and reference smacoviruses. Tree topology following the maximum likelihood (ML) approach is shown and annotated with the support of the posterior probability from the Bayesian inference approach. Smacoviruses identified in the study are shown in red color. The sequence of PkSmV1-ZM09-64 was not included in the analysis due to the unisense genome organization of the ORFs (*Rep* and *CP*).

**Figure 3:**
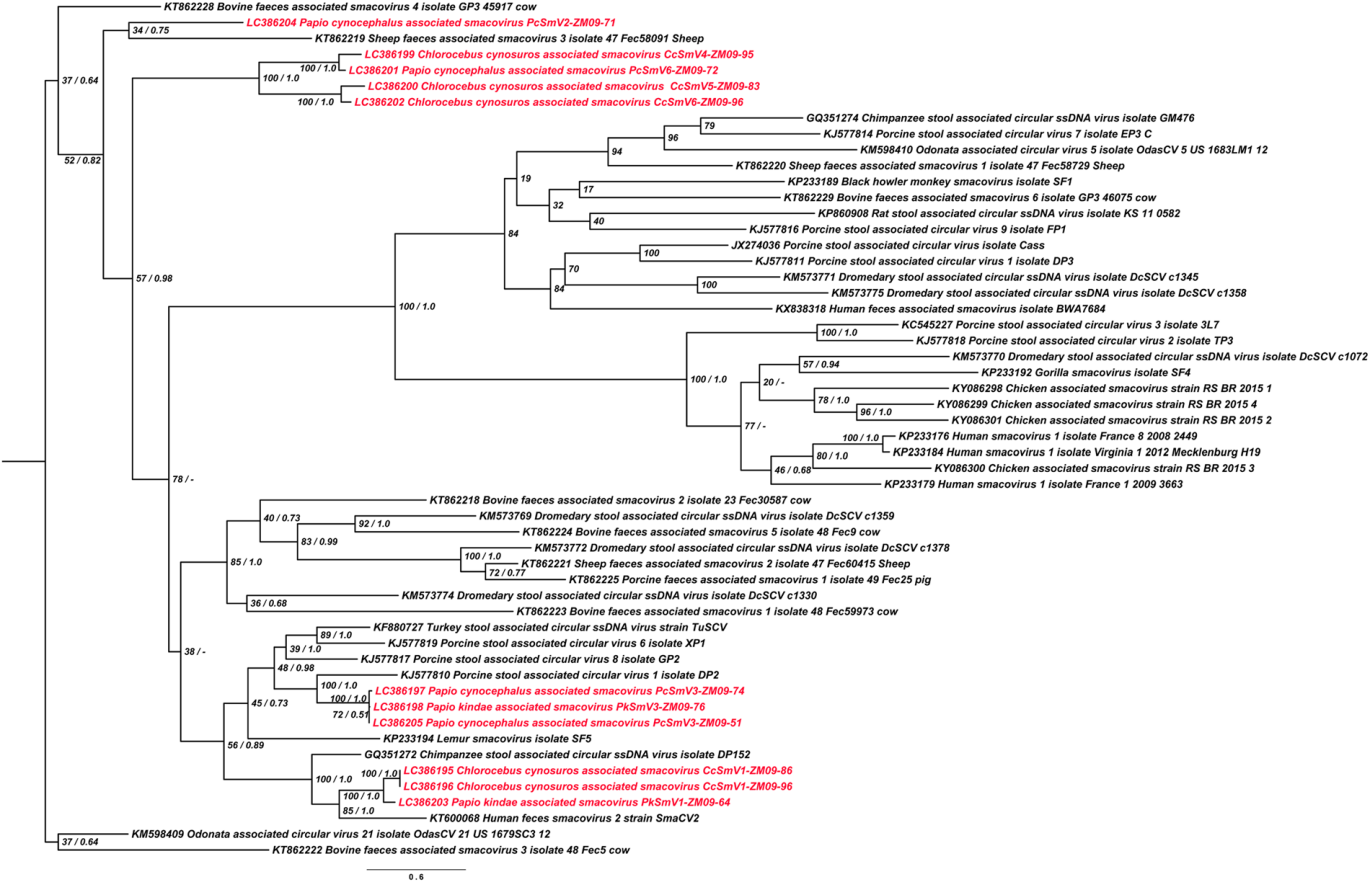
Phylogenetic tree constructed based on the CP amino acid sequences of Zambian non-human primate CRESS DNA viruses and reference smacoviruses. Tree topology following the ML approach is shown and annotated with the support of the posterior probability from the Bayesian inference approach. Smacoviruses identified in this study are shown in red color.

**Figure 4:**
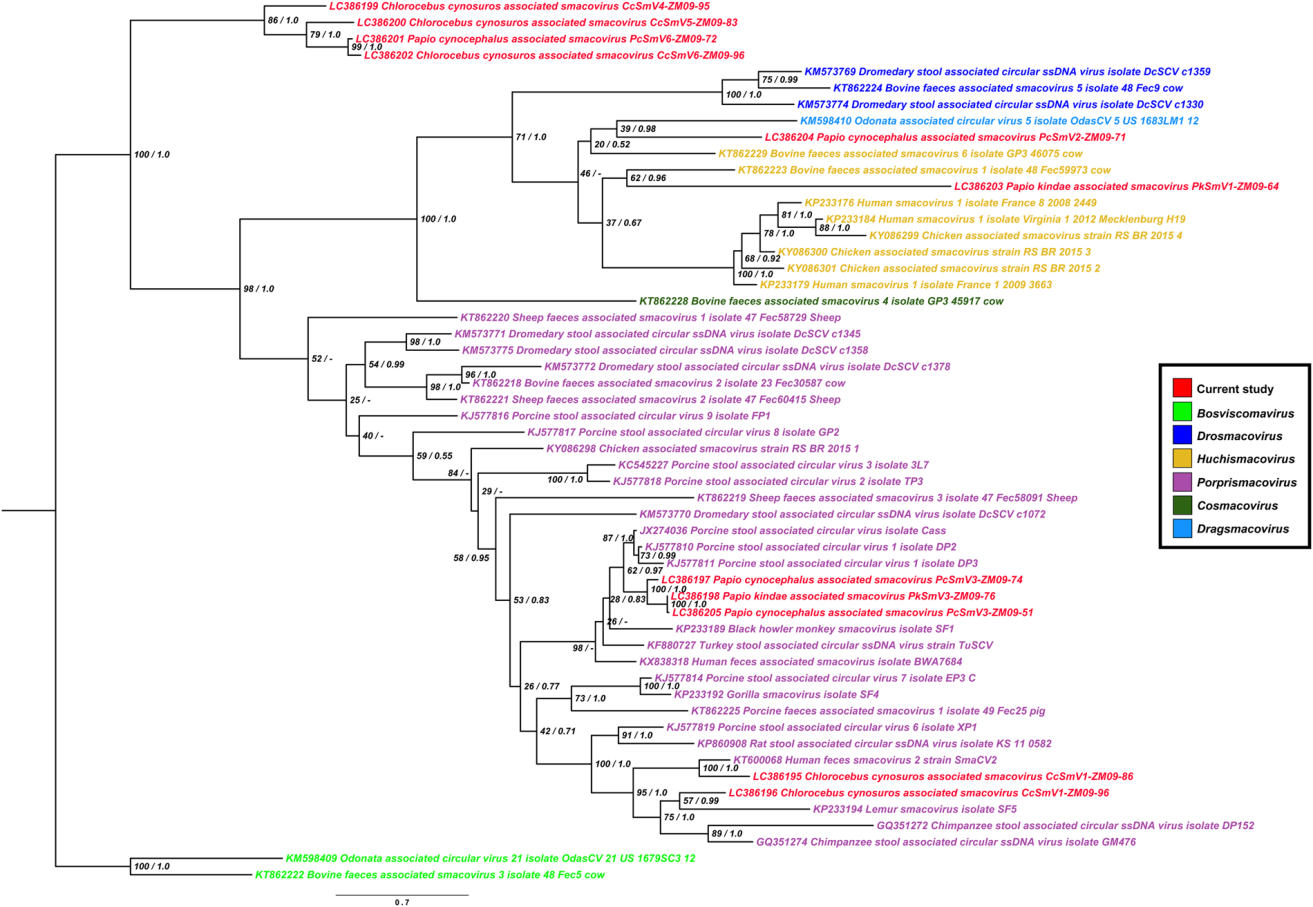
Phylogenetic tree constructed based on the Rep amino acid sequences of Zambian non-human primate CRESS DNA viruses and other known smacoviruses. Tree topology following the ML approach is shown and annotated with the support of the posterior probability from the Bayesian inference approach. Smacoviruses identified in the study are shown in red color.

Phylogenetic trees were constructed based on the analyses of the whole genome sequences (Fig. 2), and, separately, of the amino acid sequences of CP (Fig. 3) and Rep (Fig. 4) using a maximum likelihood (ML) estimation coupled with a Bayesian inference. The genome-wide phylogenetic tree revealed that ZM-SmVs, shown in red color in the tree, segregated into a cluster of previously reported smacoviruses (Fig. 2). The phylogenetic tree of Rep supported the genus assignment of the ZM-SmVs: PcSmV3-ZM09-74, PkSmV3-ZM09-76, PcSmV3-ZM09-51, CcSmV1-ZM09-86 and CcSmV1-ZM09-96 formed a cluster with members of *Porprismacovirus* and PcSmV2-ZM09-71 shared a common ancestor with members of *Huchismacovirus* (Fig. 4). CcSmV4-ZM09-95, CcSmV5-ZM09-83, PcSmV6-ZM09-72 and CcSmV6-ZM09-96 were distinct from known smacoviruses with low amino acid identities for their Rep proteins (Table 3).

Even though a clear and unambiguous congruence between the CP and Rep phylogenies was not apparent, PcSmV6-ZM09-72, CcSmV5-ZM09-83, CcSmV4-ZM09-95, and CcSmV6-ZM09-96 were clustered together in both CP and Rep trees, forming their own group (Figs 3 and 4) suggesting that they likely shared a common ancestor. PcSmV3-ZM09-51, PcSmV3-ZM09-74, and PkSmV3-ZM09-76 also consistently clustered together with porcine stool associated circular virus 1 (DP2, KJ577810) throughout the CP and Rep trees (Figs 3 and 4). In contrast, PkSmV1-ZM09-64 clustered together with CcSmV1-ZM09-86, CcSmV1-ZM09-96, human feces smacovirus 2 (SmaCV2, KT600068) and chimpanzee stool associated circular ssDNA virus (DP152, GQ351272) in the CP phylogeny (Fig. 3), whereas PkSmV1-ZM09-64 was located outside of the cluster formed by these smacoviruses in the Rep tree and was most closely related to a previously described bovine smacovirus (Fec59973, KT862223) and more distantly related to human and avian smacoviruses (Fig. 4). This phylogenetic incongruence revealed that the *CP* ORFs of PkSmV1-ZM09-64, CcSmV1-ZM09-86 and CcSmV1-ZM09-96 were derived from a common ancestor; however, the ancestor of the *Rep* gene of PkSmV1-ZM09-64 was different from that of CcSmV1-ZM09-86 and CcSmV1-ZM09-96. These findings suggested the possibility of recombination event(s) that may account for the discordant phylogenies. In addition, PcSmV2-ZM09-71 did not cluster with other ZM-SmVs throughout the constructed trees (Figs 2, 3 and 4). Taken together, these results indicated that all ZM-SmVs discovered in the study have distinct evolutionary histories and PkSmV1-ZM09-64 may have arisen from recombination.

### Recombination analysis of smacovirus genomes

**Figure 5:**
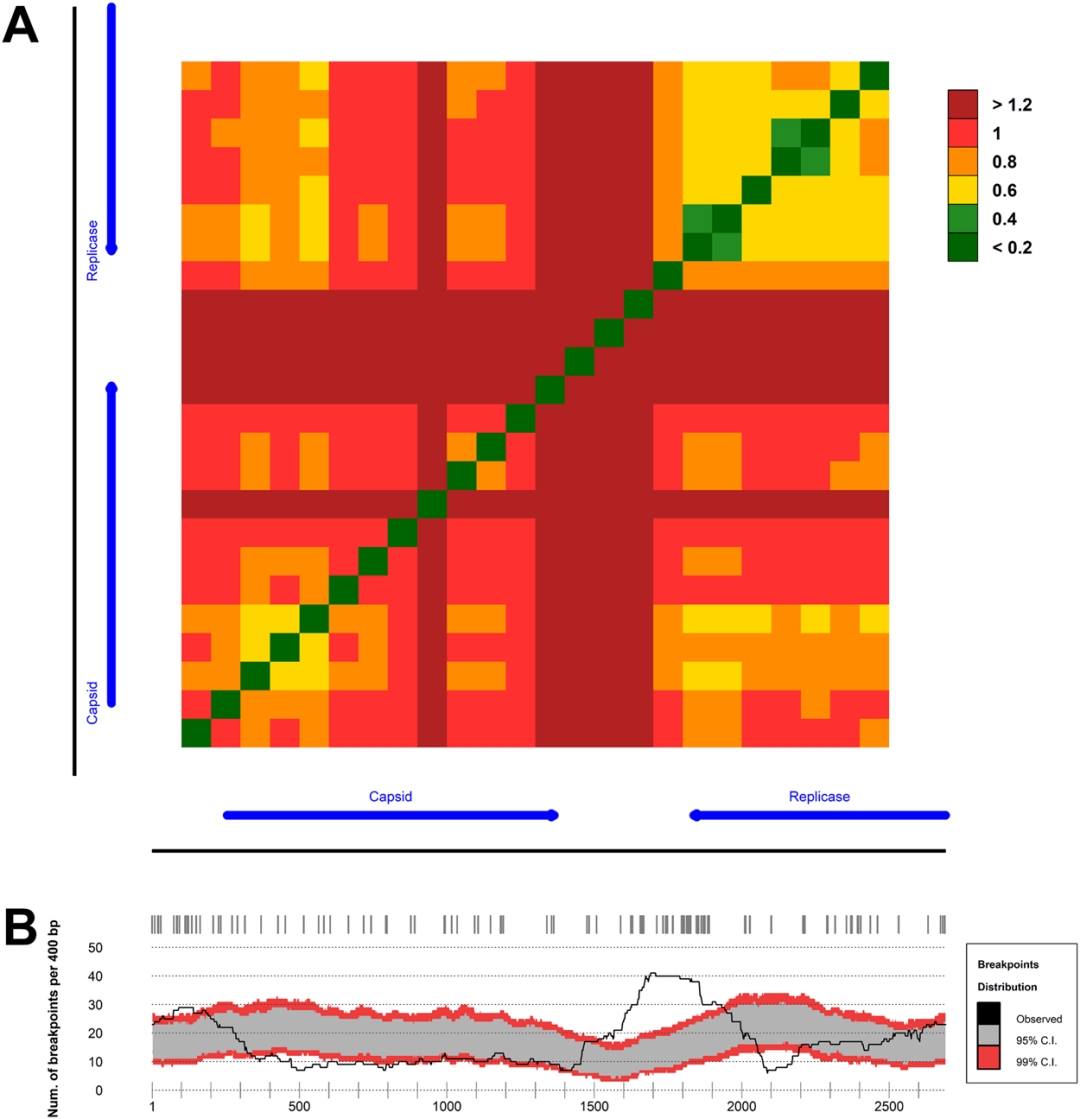
Recombination analysis of smacovirus species. (**A**) Phylogenetic compatibility matrix over the species of smacoviruses including ZM-SmVs. The respective regions in the smacoviruses alignment correspond to the normalized Robinson-Foulds distance between two neighbor-joining (NJ) trees. (**B**) Distribution of recombination breakpoints within the smacoviruses alignment. Breakpoints detected by recombination detection program (RDP) analysis are shown at top of the panel. The vertical axis is the number of recombination boundaries per window. The solid black line indicates the number of observed breakpoints. The grey and red areas indicate 95 and 99% confidence intervals of each breakpoint, respectively.

Detection of the phylogenetic incongruence between the CP and Rep phylogenies prompted us to investigate whether potential recombination sites existed in the ZM-SmV genomes. This recombination analysis revealed a region with multiple recombination breakpoints (i.e. a recombination hot spot) adjacent to the 3′-end of the *Rep* ORF (Fig. 5), which, interestingly, has also been inferred by another study^13^. These results corroborate prior studies indicating that smacoviruses increase their genetic diversity through recombination events. Two cold spots, where a recombination event is less likely to occur, were observed at the 5′-end of the *CP* ORF and the 3′-end of the *Rep* ORF. The presence of these cold spots implies functional conservation which is noteworthy in viruses as diverse as the ssDNA smacoviruses and suggests importance for these regions in the viral life cycle.

## Discussion

In the present study, sub-genomic fragments of CRESS DNA virus were initially detected in the faeces of NHPs in Zambia by metagenomic analysis. The complete genomes of eleven CRESS DNA viruses were then subsequently recovered by inverse PCR in 20% of faecal samples indicative of a high prevalence in Zambian NHPs. Although, a single CRESS DNA virus was found in nine individuals, two were found in a single Zambian malbrouck suggestive of a co-infection event (Table 1), a necessary precondition for viral recombination and emergence of new strains. Based on the genome-wide pairwise sequence identity analysis and degree of sequence divergence, these newly identified viruses could be tentatively classified as novel smacovirus species and await formal classification by the ICTV^6^. ZM-SmVs from both malbroucks and baboons formed distinct clusters within the genus *Porprismacovirus* on the phylogenetic trees, suggesting that they evolved from different ancestral progenitors further exemplifying the extent of the viral diversity.

Despite the high prevalence in Zambian NHPs, the detected ZM-SmVs showed phylogenetic divergence and there was no evidence for spread of specific ZM-SmV strains in the species of monkey and baboon NHPs we studied, which raises the question whether ZM-SmVs infect and transmit among NHPs and potentially also other species. Detection of these viruses in the NHP faecal matter suggests at least two distinct hypotheses with respect to their origin. First is that they might productively infect the NHPs; however, smacoviruses have not been identified in animal tissues^6^. Second is that they may represent ssDNA viruses ultimately derived from plants, insects or mammals, which comprise the NHP diet or from a resident microorganism of the NHP gut^11,13,20,21^. Indeed, a recent study has described high sequence similarities between smacoviral genomes and spacer sequences of a faecal archaeon, *Candidatus* Methanomassiliicoccus intestinalis, indicating a tropism of smacoviruses for archaea^22^. To date, and in common with a growing number of ssDNA and other uncultured viruses, isolates of infectious smacoviruses have not been reported. Taken together, the precise origins of the ZM-SmVs reported here remain to be established and further studies including attempts at isolation of smacoviruses are needed to characterize smacovirus infection in detail.

There was no clear congruence between the CP and Rep phylogenies for the identified CRESS DNA viruses. Specifically, PkSmV1-ZM09-64 showed clearly different phylogenetic relationships in both the CP and Rep trees. We also detected a potential recombination hot spot of breakpoints in the genome of smacovirus at the 3′-end of the *Rep* ORF providing further evidence of the importance of recombination events during the evolution of smacoviruses^13,23,24^. A recent study has also reported that the Rep of these viruses is chimeric and likely derives from recombination events that lead to intra-host lineage diversification^24^. Interestingly, the recombination analysis showed the breakpoint hot spot extended into the intergenic region between the *CP* and *Rep* ORFs. This observation has been seen in diverse ssRNA^25^ and dsDNA viruses^26^ and supposed the existence of “functionally interchangeable modules”, i.e. shuffling of the *CP* ORF by recombination may conceivably impact on virus tropism of recombinants. Our results are in agreement with the notion of recombination patterns including a mechanistic predisposition to recombination in virion-strand replication origin and recombination breakpoints which significantly tend to occur in intergenic regions or at 5′ and 3′ termini of genes rather than within the genes of ssDNA viruses^23,27^. Recombination breakpoints are known to be disfavoured within coding regions, as observed in the CP. Therefore, genes in ssDNA viruses preferentially move as modules which contain >50% of the coding region and natural selection disfavours viruses harbouring recombinant proteins which leads to the observed nonrandom distribution of breakpoint observed^27^. The modular genetic exchange by recombination within non-coding regions have also been previously implicated in the emergence of new viral strains^28,29^. Whether these, or related phenomena, exist for recombinant smarcoviruses warrants further study.

PkSmV1-ZM09-64 showed a unisense genome organization of *CP* and *Rep* ORF similar to previously reported CRESS DNA virus, PigSCV (JQ023166) (Fig. 1)^8^. The precise reasons underlying this ORF organization by these CRESS DNA viruses remain unclear. It is possible that this genome organization may have arisen from errors during recombination event between ancestral viruses which led to a unidirectional ORF organization instead of the more common ambisense bidirectional genome organization evident in the majority of known smacoviruses.

In conclusion, our studies indicate the presence of previously unrecognized CRESS DNA viruses in the NHP virome and provide further evidence of the extent of the genetic diversity of DNA viruses in primates.

## Materials and Methods

### Ethical statement and sample collection

All animal experiments were approved by the then Zambia Wildlife Authority (ZAWA), now the Department of National Parks and Wildlife, Ministry of Tourism and Arts and performed in accordance with the relevant guidelines and regulations (certificate no. 2604). Tissue and faecal samples were collected from NHPs (*Chlorocebus cynosuros*, n = 25; *Papio spp*., n = 25) in Mfuwe District in 2009 and used for different research projects as reported previously^30,31^. For NHP species typing, the mitochondrial *cytb* gene was amplified and sequenced from genomic DNA extracted from spleen tissues of the NHPs, as described elsewhere^31^.

### Metagenomic analysis using high-throughput sequencing

Viral nucleic acids were extracted from the pooled faecal suspensions as described previously^32^, and double-stranded cDNA was synthesized with the PrimeScript Double Strand cDNA Synthesis kit (Takara BIO, Shiga, Japan). Sequencing libraries were prepared with Nextera XT DNA Sample Preparation kit (Illumina, San Diego, CA) and sequenced on the Illumina MiSeq platform (Illumina). The obtained reads were compared against NCBI NT/NR database as described previously^33^. The sequence reads related to smacoviruses were *de novo* assembled to contigs using CLC Genomics Workbench software (CLC bio, Aarhus, Denmark).

### PCR screening, whole genome sequencing, and genome annotation

Based on the nucleotide sequences of the generated contigs, six different pairs of primer sets were designed for PCR screening with GENETYX software (GENETYX, Tokyo, Japan) (Supplementary Table S1). DNA was extracted from faecal samples for each individual NHP with the High Pure Viral Nucleic Acid kit (Roche Diagnostics, Mannheim, Germany) and PCR screened for putative smacoviruses with Tks Gflex DNA Polymerase (TAKARA BIO). PCR products were sequenced and the sequences were used to design additional primers for the complete genome amplification of CRESS DNA viruses by inverse PCR. The amplicons were subsequently cloned into a pCR4-Blunt-TOPO vector (Invitrogen; Thermo Fischer Scientific, Waltham, MA) and sequenced by a primer walking strategy. The whole circular genome of each CRESS DNA virus was assembled with Phred and Phrap^34^ with quality scores >30 in all assembled nucleotide positions and annotated using Geneious^35^. The pairwise identity among sequences was calculated with Sequence Demarcation Tool (SDT) v1.2^36^.

### Phylogenetic analysis

The complete genome nucleotide sequences and predicted amino acid protein sequences were aligned with MAFFT using the algorithm FFT-NS-i^37^. To infer the phylogenetic relation between sequenced and available samples maximum likelihood (ML) approaches with IQ-TREE v1.6.5^38^ were used to determine the best substitution model, infer the topology and the branch support with a bootstrap of 1,000 repetitions. Additionally, Bayesian inference approaches with MrBayes v3.2.6^39^ were used to search for the best substitution model and estimate the posterior probability of the inferred branches with chains of one million states. Three phylogenetic trees were inferred in this study for the whole genome nucleotide sequences (Fig. 2), and the amino acid sequences of CP (Fig. 3) and Rep (Fig. 4). The tree topology of the ML approach was used and annotated with the support of the posterior probability from the Bayesian approach.

### Recombination analysis

The genome multiple sequence alignment was assessed for evidence of recombination events by the suite of methods in the recombination detection program (RDP) v.4.58^40,41^. Detected recombination events required statistical support *p* < 0.01 and the distribution of recombination breakpoints were analyzed with a sliding window of 400 nucleotides and one nucleotide step, with 1,000 permutations for estimating the statistical support of the breakpoint distribution. To assess the effects of the recombination events on the phylogenetic relationships among sequences, a compatibility matrix was built^25^, where the compatibility of two windows with 300 nucleotides from a sliding window and 100 nucleotides per step is defined as the normalized Robinson-Foulds distance^42^ between the corresponding neighbor-joining phylogenetic trees under Tamura-Nei substitution model. The compatibility reflects how similar are the inferred phylogenies for any two genome windows ranging between 0 (identical topologies) to 1 (completely dissimilar topologies).

## Supplementary information

Supplementary Table S1

## Data Availability

The whole genomes of the identified viruses in this study were submitted to the GenBank/EMBL/DDBJ database under accession numbers of LC386195-LC386205.
