# Publisher Correction: Discovery and genetic characterization of diverse smacoviruses in Zambian non-human primates

**DOI:** 10.1038/s41598-019-44438-2

**Published:** 2019-06-06

**Authors:** Paulina D. Anindita, Michihito Sasaki, Gabriel Gonzalez, Wallaya Phongphaew, Michael Carr, Bernard M. Hang’ombe, Aaron S. Mweene, Kimihito Ito, Yasuko Orba, Hirofumi Sawa

**Affiliations:** 1 Division of Molecular Pathobiology, Research Center for Zoonosis Control, Hokkaido University, Sapporo, 001-0020 Japan; 2 Division of Bioinformatics, Research Center for Zoonosis Control, Hokkaido University, Sapporo, 001-0020 Japan; 3 Global Institution for Collaborative Research and Education (GI-CoRE), Hokkaido University, Sapporo, 001-0020 Japan; 4 National Virus Reference Laboratory, School of Medicine, University College Dublin, Belfield, Dublin 4 Ireland; 5 Department of Paraclinical Studies, School of Veterinary Medicine, University of Zambia, PO Box 32379, Lusaka, 10101 Zambia; 6 Department of Disease Control, School of Veterinary Medicine, University of Zambia, PO Box 32379, Lusaka, 10101 Zambia; 7 Africa Centre of Excellence for Infectious Diseases of Humans and Animals, University of Zambia, P.O. Box 32379, Lusaka, 10101 Zambia; 8 Global Virus Network Affiliate, University of Zambia, P.O. Box 32379, Lusaka, 10101 Zambia; 9 Global Virus Network, 801 W. Baltimore St., Baltimore, MD 21201 USA

Correction to: *Scientific Reports* 10.1038/s41598-019-41358-z, published online 8 April 2019

The original PDF version of this Article contained an error in the publication date, which was incorrectly given as 25th March 2019. This error has now been corrected in the PDF version; the HTML version was correct from the time of publication.

