# Supplementary material for: Discovery and genetic characterization of diverse smacoviruses in Zambian non-human primates
Source: Sci Rep. 2019 Apr 8;9:5045. doi: 10.1038/s41598-019-41358-z (PMC6453971; doi:10.1038/s41598-019-41358-z)
Supplement: Supplementary file 1 — Supplementary Table S1 [file 41598_2019_41358_MOESM1_ESM.docx]

Supplementary Information

**Discovery and genetic characterization of diverse smacoviruses in Zambian non-human primates**

Paulina D. Anindita, Michihito Sasaki, Gabriel Gonzalez, Wallaya Phongphaew, Michael Carr, Bernard M. Hang’ombe, Aaron S. Mweene, Kimihito Ito, Yasuko Orba, Hirofumi Sawa

Table S1. List of primers used to examine the PCR prevalence of detected CRESS DNA viruses

| **No.** | **Primer name** | **Sequences (5’ to 3’)** |
| --- | --- | --- |
| 1. | ssDNAV-1F | ATGGTGCCTCTAGGGTCTATTATCC |
| 2. | ssDNAV-1R | AATCTCCGACAATCGARGGCTGAC |
| 3. | ssDNAV-2F | GGTCAGCGTACTGTTGTTCAGGAGA |
| 4. | ssDNAV-2R | TGATAAAAGACTCATCACGCCATTC |
| 5. | ssDNAV-3F | CCGATCGTTTATTCTGTTGTCTCTG |
| 6. | ssDNAV-3R | GAAGAGCATAATACATGCTGAACTG |
| 7. | ssDNAV-4F | GTCCGTATCCTGCTTTGCCTACC |
| 8. | ssDNAV-4R | AGCGCCAGGACGATTAATAGCAAC |
| 9. | ssDNAV-5F | CGATTATGCTTTGTCGACCCAATCC |
| 10. | ssDNAV-5R | AAATAGGACATCCCCGTTTCAGG |
| 11. | ssDNAV-6F | GTAATTCTTATCATGCGACAATGCC |
| 12. | ssDNAV-6R | GGAGAAGCATTACCACGCATACCC |
